# Random Access for Underwater Acoustic Cellular Systems

**DOI:** 10.3390/s18020432

**Published:** 2018-02-01

**Authors:** Rothna Pec, Mohammed Saquib Khan, Muhammad Asim, Yong Soo Cho

**Affiliations:** School of Electrical and Electronics Engineering, Chung-Ang University, Seoul 156-756, Korea; pecrothna@yahoo.com (R.P.); snhk02@gmail.com (M.S.K.); muhammad3428@gmail.com (M.A.)

**Keywords:** underwater acoustic cellular system, random access, ambiguity function, Doppler environment

## Abstract

In this paper, a random access preamble (RAP) design technique for underwater acoustic cellular systems is proposed. After showing that the conventional RAP used in long term evolution (LTE) systems is not appropriate for underwater acoustic cellular systems, two different types of RAPs (RAP 1 and RAP 2) are proposed to detect the identity of underwater equipment/nodes (UEs) and estimate the time delay between a UE and an underwater base station (UBS) at the physical layer. RAP 1 is generated using a Zadoff-Chu (ZC) sequence where the identity of the UE is mapped to its root index, whereas RAP 2 is generated using a linear frequency modulation (LFM) waveform where the identity of the UE is mapped to its frequency sweeping parameter and frequency shifting parameter. Ambiguity functions (AFs) and cross-ambiguity functions (CAFs) of RAP 1 and RAP 2 are derived to investigate their correlation properties under the effect of time delay and Doppler shift. The performance of RAP detection is investigated by analyzing the detection probabilities and false alarm probabilities of RAP 1 and RAP 2 in a Doppler environment. By evaluating the performances of RAP 1 and RAP 2 in various situations, it is concluded that RAP 2 is more suitable for underwater acoustic cellular systems. The AF and CAF analytically obtained in this paper are shown to be similar to those obtained using experimental data.

## 1. Introduction

During the last decade, interest in the study of underwater communication systems for ocean observation has increased owing to their many scientific, military, and commercial applications [1]. These applications range from tactical surveillance to the study of marine life, and include autonomous underwater vehicle (AUV) communication, pollution monitoring, oil extraction monitoring, and aquaculture monitoring [2,3]. Recently, the number of applications requiring high bandwidth has been increasing because AUVs and sensors are being deployed to gather important data such as real-time videos, environmental, and security data.

However, the propagation of sound in oceans is known to be very complex [4]. Owing to the reflections on the surface of the ocean and refractions caused by depth-varying sound speed, sound tends to propagate through multipath trajectories; the temporal variability of the ocean combined with the low sound speed in water may induce significant Doppler shifts. Consequently, the channel is affected by time and/or frequency dispersion. The attenuation of acoustic signals is also known to increase with frequency and range. The available bandwidth of an underwater channel is limited to the low frequency region and depends on both the transmission range and frequency.

Pioneering work on underwater acoustic cellular systems was performed in [5] because the available bandwidth in underwater is severely limited. In these studies, the concept of reusing frequencies widely used in terrestrial cellular systems was utilized to increase both the coverage and capacity. Neighboring cells had different sets of carrier frequencies to avoid interference and the cells sufficiently far apart were operated at the same frequency. The design technique of an underwater cellular network, such as the determination of cell size and frequency reuse pattern, was investigated. In [6], the capacity of an underwater acoustic cellular system that uses orthogonal frequency-division multiplexing (OFDM) was analyzed. Using the frequency-dependent absorption characteristic of underwater acoustic channel, the concept of multiple architectural layers was proposed wherein higher/lower frequencies were repeated more/less frequently over the deployment region. It was shown that the performance can be improved by using multiple architectural layers wherein a different reuse number is assigned to each subcarrier. The capacity of an underwater acoustic cellular system based on OFDM was shown to be better than that of a single carrier system. A code-division multiple access (CDMA)-based underwater acoustic cellular system and cellular underwater wireless optical CDMA system were proposed in [7,8,9].

Similar to terrestrial cellular systems, an initial access procedure is required for an underwater equipment/node (UE) in an underwater acoustic cellular system to establish a communication link with an underwater base station (UBS). In order to establish a communication link, the UE should perform downlink synchronization and cell searching by receiving synchronization and broadcasting signals [10]. After obtaining downlink (DL) synchronization and system information including parameters specific to random access, the UE performs random access preamble (RAP) transmission [11,12]. The UE selects one of the RAPs and transmits it using the time-frequency resources indicated by the system information. The main purpose of RAP transmission is to inform the UBS regarding the presence of the UE and to allow the UBS to estimate the time delay between UBS and UE. The process of estimating the delay is especially important in underwater acoustic cellular systems because underwater channels depend strongly on frequency and distance in contrast to terrestrial cellular systems. In other words, the propagation loss owing to absorption increases significantly with frequency and distance. Thus, in underwater acoustic systems, it will be beneficial to allocate a high frequency band to the nearby UE whereas a low frequency band is allocated to far UE. The capacity of the system can be maximized using a channel allocation scheme that utilizes the location of the UE.

In this paper, a design technique of RAP for underwater acoustic cellular systems is proposed. First, it is shown that the conventional RAP used in long term evolution (LTE) systems is not appropriate for underwater acoustic cellular systems because acoustic waves propagate in water at a very low speed, which is five orders of magnitude smaller than the propagation speed of radio waves in the air. Subsequently, two different types of RAPs (RAP 1 and RAP 2) are proposed to detect the identity of UE and estimate the time delay between the UBS and UE at the physical layer. RAP 1 is generated using a Zadoff-Chu (ZC) sequence where the identity of the UE is mapped to its root index, whereas RAP 2 is generated using a linear frequency modulation (LFM) waveform where the identity of the UE is mapped to its frequency sweeping parameter and frequency shifting parameter. Ambiguity functions (AFs) of RAP 1 and RAP 2 are analyzed to investigate their correlation properties under the effect of time delay and Doppler shift. Subsequently, the definition of AF is extended to a cross-ambiguity function (CAF) to analyze the cross-correlation properties of the proposed RAPs with different IDs of UE under the effect of time delay and Doppler shift. The performance of RAP detection is investigated by analyzing the detection probabilities and false alarm probabilities of the proposed RAPs in a Doppler environment. By evaluating the performances of the proposed RAPs in various situations, it is concluded that RAP 2 is more suitable for underwater acoustic cellular systems. The AF and CAF analytically obtained in this paper are shown to be similar to those obtained using experimental data.

The rest of this paper is organized as follows: in Section 2, we provide an overview of an underwater acoustic cellular system including underwater frequency allocation map and downlink frame structure. In Section 3, RAP 1 and RAP 2 are proposed for underwater acoustic cellular systems after reviewing the conventional technique used in LTE cellular systems. In Section 4, we analyze the detection probability and false alarm probability of the proposed RAPs in a Doppler environment. In Section 5, the experimental results are compared with the analytical results obtained in Section 3. The conclusions drawn are presented in Section 6.

## 2. System Model

**Figure 1:**
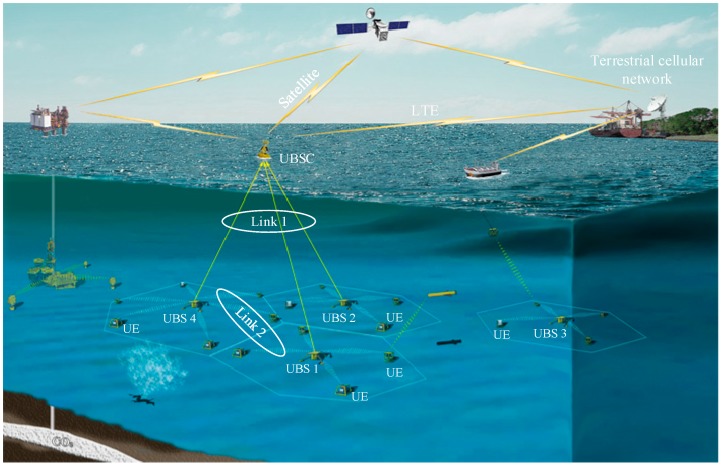
Conceptual view of an underwater acoustic cellular system.

**Figure 2:**
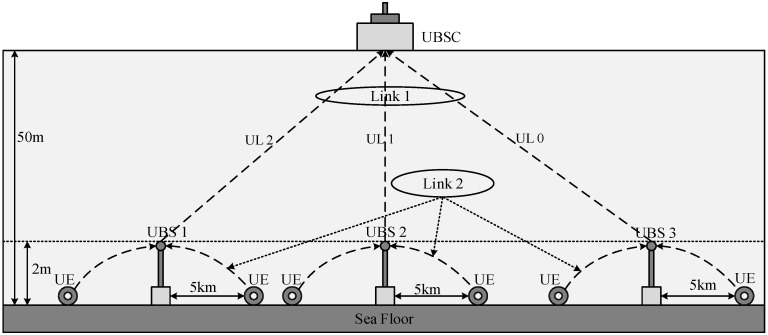
Cross-section of an underwater cellular system.

**Figure 3:**
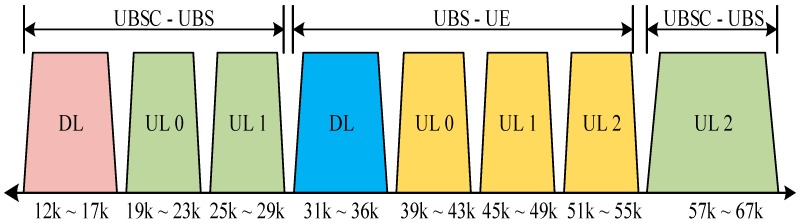
Underwater frequency allocation map.

Figure 1 shows the conceptual view of an underwater acoustic cellular system connected to terrestrial cellular networks via LTE or satellite. Figure 2 shows the cross-section of an underwater acoustic cellular system consisting of two different links (Link 1 and Link 2). In Link 2, a group of UEs are connected to the serving UBS located on the sea floor. A UE can be connected to one or more UBSs by means of the wireless acoustic Link 2 when the UE is in the handover region. UBSs are connected to a UBS controller (UBSC) or surface buoys via a wireless acoustic backbone link (Link 1). Figure 3 shows an example of a frequency allocation map for the underwater acoustic cellular system in Figure 2. As shown in Figure 3, different frequency bands are used for Link 1 and Link 2 to avoid interference between the links. Moreover, a frequency division duplex (FDD) scheme is used for DL and UL channels in both Link 1 and Link 2. In Link 1, a downlink channel and three uplink channels (UL 0, UL 1 and UL 2) are assigned. The lower frequency bands are assigned to Link 1 (except UL 2) because a backbone link is more important. The lowest frequency band is assigned to the DL channel because it is common to all the UBSs. UL 2 is assigned to a special node requiring high data rate (e.g., AUV), operating near the UBSC. Furthermore, a downlink channel and three uplink channels are assigned to Link 2. Similar to Link 1, the lowest frequency band in Link 2 is assigned to the DL channel because it is common to all the UEs. Moreover, the lowest frequency band (UL 0) in the UL is assigned to far UEs whereas the highest frequency band (UL 2) in the UL is assigned to nearby UEs. The frequency band allocation is performed in the initialization stage using the information of the estimated time delays (locations) of the UEs (or UBSs) to maximize system capacity. For all the UEs in Link 2 or all the UBSs in Link 1, UL 0 is used as a common channel for RAP transmission in the initialization stage. The channel bandwidth and guard band are chosen in accordance with the characteristics of the acoustic transducer and hydrophone in the DL or UL. Although various transmission/multiplexing schemes such as single carrier, orthogonal frequency-division multiplexing (OFDM), CDMA, and single-carrier frequency-division multiple access (SC-FDMA) can be used for channels in Link 1 and Link 2, an OFDM-based system is considered in this paper because it can cope with the frequency-selective fading caused by a long multipath propagation in an underwater acoustic channel, using a simple equalizer [13]. Various inter-cell interference mitigation techniques such as fractional frequency reuse, soft frequency reused, and dynamic channel allocation can be used in adjacent cells to reduce the interferences in Link 2 [14].

**Figure 4:**
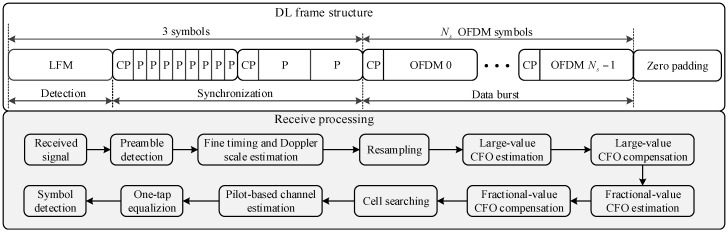
DL frame structure and receiver processing.

Figure 4 shows the example of a DL OFDM frame structure for Link 1 and Link 2 in Figure 2. A frame consists of a detection preamble, synchronization preamble, and OFDM symbols for data burst [13,14]. An LFM waveform is used for preamble detection because it is known to be robust to Doppler shifts. A repetitive pattern of ZC sequence is used for synchronization and cell searching owing to its good correlation property in time and frequency domains [15]. From the received synchronization preamble, we estimate the fine timing value and Doppler scale factor. The Doppler scaling effect on the received signal is compensated by a resampling process with the estimated Doppler scale factor. Large and small values of carrier frequency offset (CFO) are estimated using 2-repetitive and 8-repetitive patterns of a ZC sequence, respectively. After CFO compensation, a serving UBS is selected. Subsequently, a time-varying channel is estimated by pilots inserted in each OFDM symbol. Finally, transmitted data are recovered with frequency-domain equalizers obtained using the pilots. In this paper, we will assume that the downlink synchronization and cell searching process is completed, and we focus on the design of RAP using UL 0 channel.

## 3. RAP Design

In this section, two different types of RAPs for underwater acoustic cellular systems are proposed after reviewing the conventional technique used in LTE cellular systems. The design of an LTE-based RAP is described first owing to its similarity to our study and simplicity at the receiver side (low computational complexity for RAP detection).

### 3.1. LTE-Based RAP

The LTE RAP is constructed using a prime-length ZC sequence. The sequence is appended by a cyclic prefix (CP) to absorb both the round trip delay (RTD) and maximum delay spread (MDS) [11]. The prime length is chosen to maximize the number of RAPs with good correlation properties. However, the time-frequency specification of the LTE RAP does not match that of the underwater acoustic channel owing to a large discrepancy in the propagation speed and operational channel bandwidth. Since a straightforward application of the LTE RAP for underwater cellular systems is not possible, we attempt to modify the parameters of the LTE RAP according to the characteristics of the underwater acoustic channel.

The LTE RAP occupies a channel bandwidth of 1.08 MHz with the minimum sequence duration of 800 µs. The RAP slot duration (RAP format 0) including CP interval, sequence duration, and guard time interval is 1 ms for the smallest cell coverage (radius of 14 km). The lower bound of the sequence length must satisfy the following condition to facilitate unambiguous round-trip time estimation for a UE at the cell edge [11]:

(1) $$T_{SEQ}\geq \frac{2C_{R}}{v_{c}}+\delta_{\max}$$

where *C_R_*, $v_{c}$, and $\delta_{\max}$ denote the cell radius, sound speed, and MDS, respectively. For an LTE-based RAP in an underwater channel environment, the sequence length $T_{SEQ}$ must be greater than or equal to 6.8 s when the cell radius, sound speed, and MDS are 5 km, 1500 m/s, and 132 ms, respectively. A sequence length larger than the lower bound value in (1) must be chosen to guarantee coverage. If a sequence length of 7 s is selected for a cell radius of 5 km, the corresponding subcarrier spacing of the LTE-based RAP will be 0.1429 Hz, which is very small compared to that used in terrestrial communication systems (i.e., 1.25 kHz). However, small subcarrier spacing and long symbol duration are not favorable to underwater channels because the doubly-selective characteristic of an underwater acoustic channel destroys the orthogonality of the RAP. The RAP is also very sensitive to Doppler shift, resulting in not only inter-channel interference (ICI) caused by a fractional CFO but also the time ambiguity caused by an integer CFO. The time ambiguity results in an error in distance estimation, which is up to *T_SEQ_* × *ν_c_* = 7 × 1500 = 10.5 km for a sequence duration of 7 s.

In the LTE system, many RAPs are generated from a single ZC sequence by changing the values of cyclic shift index [11]. Therefore, cyclic shift dimensioning is important because it limits the number of RAPs for a selected root index. The lower bound of the cyclic shift, *N_CS_*, is given by:

(2) $$N_{CS}\geq \left\lceil \left(\frac{2C_{R}}{v_{c}}+\delta_{\max}\right)\frac{N_{ZC}}{T_{SEQ}}\right\rceil +N_{0}$$

For a sequence length, *N_ZC_*, of 499 and a zero-value of guard sample, *N*_0_, *N_CS_* ≥ 485. The number of possible RAPs generated by a ZC sequence with a root index is given by $\left\lfloor N_{ZC}/N_{CS}\right\rfloor =\left\lfloor 499/485\right\rfloor =1$. Therefore, a ZC sequence can generate only one RAP in underwater acoustic channels.

One of the advantages of using LTE-based RAP is the simplicity in the RAP detection process, which is composed of the following three steps: (1) the received RAP is transformed to the frequency domain via discrete Fourier transform (DFT); (2) the frequency-domain signal is multiplied by a conjugated version of the base sequence before transforming it to the time domain via inverse DFT (IDFT); (3) the power delay profile is computed and compared with a threshold value for RAP detection and time delay estimation. An FFT/IFFT operation with a size approximately the same as the sequence length is usually used to reduce the complexity of DFT.

In summary, the LTE-based RAP is not appropriate for underwater acoustic cellular systems because it has a long symbol duration leading to high time selectivity, a small subcarrier spacing leading to CFO sensitivity, and a large time ambiguity leading to a small number of possible RAPs, although the computational complexity for RAP detection is small.

### 3.2. Proposed RAP

**Figure 5:**
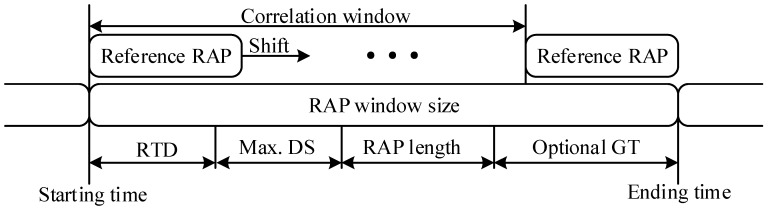
RAP detection process.

In order to overcome the limitations of the LTE-based RAP, we propose two different RAPs that are more suitable for underwater acoustic channels. The proposed RAPs, which are generated and detected in the time domain, have short symbol duration, compared with the LTE-based RAP. The proposed RAP is transmitted in the lowest frequency band (UL0), which is common to all the UEs in Link 2 (or UBSs in Link 1) in the initialization stage. A UE transmits an RAP at the predefined time slot obtained by decoding the downlink broadcast information. As shown in Figure 5, RAP detection is performed at the receiver side by correlating the received signal with local (reference) RAPs within the RAP window. The output of linear-shift (not cyclic-shift) correlation is compared with a threshold value to detect the RAP. The RAP window should be sufficiently long to cover RTD, MDS, and RAP length. Additional guard time (GT) might be configured if necessary.

#### 3.2.1. Proposed RAP 1

RAP 1 is generated using a prime-length *ZC* sequence with a root index *u_c_* where the ID of a UE, *c*, is mapped. RAP 1 of sequence length *N_ZC_* is defined in the discrete-time domain as follows:

(3) $$x_{\mathrm{ZC}}^{c}\left(n\right)=x_{u_{c}}\left(n\right)q\left(n\right),\;0<u_{c}<N_{ZC}$$

The *ZC* sequence, *x_u_*(*n*), with a root index *u* and rectangle function, *q*(*n*), are defined as:

(4) $$x_{u}\left(n\right)=e^{j\frac{\pi u}{N_{ZC}}n\left(n+1\right)},\;q\left(n\right)=\{\begin{array}{ll} 1, & n=0,1,\cdots ,N_{ZC}-1\\ 0, & \mathrm{otherwise} \end{array}$$

Here, the length of *ZC* sequence is chosen as a prime number because it maximizes the number of root indices, thus achieving good correlation property.

In order to analyze the properties of the proposed RAP in terms of time delay and Doppler shift, its AF is analyzed. The AF of RAP 1 with ID *c* is given in the discrete-time domain as follows:

(5) $$\begin{array}{cc} A_{ZC}^{c}\left(n,\varepsilon \right) & =\frac{1}{N_{ZC}}\sum_{m=-\infty }^{\infty }x_{ZC}^{c}\left(m\right){\left(x_{ZC}^{c}\left(m-n\right)\right)}^{*}e^{j\frac{2\pi \varepsilon }{N_{ZC}}m}\\ & =\frac{1}{N_{ZC}}\sum_{m=-\infty }^{\infty }x_{u_{c}}\left(m\right)x_{u_{c}}^{*}\left(m-n\right)q\left(m\right)q\left(m-n\right)e^{j\frac{2\pi \varepsilon }{N_{ZC}}m}\\ & =\rho_{ZC}\left(n\right)e^{j\pi \frac{\left(nu_{c}+\varepsilon \right)N_{ZC}+\varepsilon \left(n-1\right)}{N_{ZC}}}\{\begin{array}{cc} 1, & \left(nu_{c}+\varepsilon \right)\mathrm{mod}N_{ZC}=0\\ \frac{\sin c\left(\rho_{ZC}\left(n\right)\left(nu_{c}+\varepsilon \right)\right)}{\sin c\left(\frac{nu_{c}+\varepsilon }{N_{ZC}}\right)}, & \mathrm{otherwise} \end{array} \end{array}$$

where *ε = f_D_τ*. Here, *f_D_* and *τ* denote the Doppler shift and duration of RAP, respectively. Furthermore, *a*mod*b* denotes the remainder of division and sinc(*x*) denotes the normalized sinc function (=sin(*πx*)/*πx*). Further, $\rho_{ZC}\left(n\right)=1-{\left|n\right|/N}_{ZC}$. For |*n*| ≥ *N_ZC_*, $A_{ZC}^{c}\left(n,\varepsilon \right)=0$. As shown in (5), the magnitude at the origin (*n*_0_ = 0, *ε* = 0) is one. For a small value of *ε* (compared with *N_ZC_*), the magnitude of AF at the correct timing can be approximated as $\left|A_{ZC}^{c}\left(0,\varepsilon \right)\right|\approx \left|\sin c\left(\varepsilon \right)\right|$, which is a sinc function of *ε*. This result signifies that the magnitude at the correct timing decreases as the Doppler shift increases. It becomes zero when the value of *f_D_τ* is an integer number. Therefore, the magnitude of AF at the correct timing decreases significantly when the Doppler shift is large. The position of side peaks, which may contribute to the RAP detection with a large timing error, can also be predicted using (5). The position (*n_i_*) of the *i-*th (i>0) significant side peak can be obtained as *n_i_u_c_* ≈ *α_i_N_ZC_* (−*N_ZC_* < *n_i_* < *N_ZC_*), i.e., the side peak occurs at *n_i_* when the product of *n_i_* and *u_c_* is given by an integer value close to the product of the sequence length and an arbitrary integer value *α_i_*. The maximum value of side peak occurs when (*n_i_u_c_* + *ε*)mod*N_ZC_* = 0, with the value of *ρ_ZC_*(*n_i_*) at *n_i_*. The term (*n_i_u_c_* + *ε*)mod*N_ZC_* becomes zero only when *ε* is a non-zero integer value. For example, if *u_c_* = 5, side peaks occur at *n*_1_ = ±100 and *n*_2_ = ±200 because 100 × 5 = 500 and 200 × 5 = 1000 are the closest integer values to 499 and 998, respectively. The maximum values of side peak occur when the values of *ε* are equal to −1 and −2, because (500−1)mod499 = 0 and (1000−2)mod499 = 0, respectively. In this case, *ρ_ZC_*(100) = 0.8 and *ρ_ZC_*(200) = 0.6.

**Figure 6:**
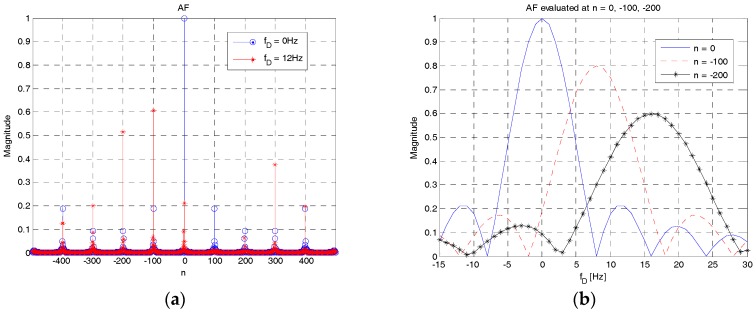
AF of RAP 1. (**a**) AF vs. time delay; (**b**) AF vs. Doppler shift.

Figure 6 shows the AF of RAP 1 with a root index of 5 as a function of time delay (Figure 6a) and Doppler shift (Figure 6b). As shown in Figure 6a, the magnitude of AF is 1 at the correct timing (*n* = 0) and decreases in a sinc pattern as *n* increases or decreases, when the Doppler shift is 0 (circle). Significant side peaks occur at |n|={100,200,300,400} when the Doppler shift is 12 Hz (star). When *f_D_* = 0 Hz, the magnitudes of side peaks are smaller than 0.2. However, when *f_D_* = 12 Hz, the magnitude at the correct timing is reduced to approximately 0.2 and the side peaks become larger, leading to RAP detection with a large timing error. Figure 6b shows the behavior of AF evaluated at three different time indices, n = {0, −100, −200}, when the Doppler shift varies. As shown in this figure, $\left|A_{ZC}^{c}\left(0,\varepsilon \right)\right|$, $\left|A_{ZC}^{c}\left(-100,\varepsilon \right)\right|$ and $\left|A_{ZC}^{c}\left(-200,\varepsilon \right)\right|$ have sinc shapes decreasing from the maximum values of 1, 0.8, and 0.6, respectively. The largest magnitudes of $\left|A_{ZC}^{c}\left(-100,\varepsilon \right)\right|$ and $\left|A_{ZC}^{c}\left(-200,\varepsilon \right)\right|$ occur at the first and second nulling points in $\left|A_{ZC}^{c}\left(0,\varepsilon \right)\right|$, i.e., at the Doppler shifts of 8 Hz and 18 Hz, respectively. The cross-over point between $\left|A_{ZC}^{c}\left(0,\varepsilon \right)\right|$ and $\left|A_{ZC}^{c}\left(-100,\varepsilon \right)\right|$ occurs near the Doppler shift of 4.13 Hz, with the magnitude of 0.61. Notably, the magnitudes of other side peaks including the peak at the correct timing are less than 0.2 when the Doppler shift is 4.13 Hz. 

These simulation results confirm that RAP 1 is sensitive to Doppler shift; the magnitude of AF at the correct timing is reduced and side peaks increase in a Doppler environment. From this result, it can also be concluded that the approach widely used in LTE-based systems to increase the number of RAPs, i.e., cyclic-shifting a ZC sequence, is not suitable for carrying the IDs of different UEs in underwater acoustic cellular systems owing to high auto-correlation in RAP 1 in a Doppler environment.

Subsequently, in order to analyze the cross-correlation properties of the proposed RAPs with the different IDs of UEs under the effect of time delay and Doppler shift, the definition of AF is extended to CAF in this paper. The discrete-time CAF of RAP 1 with two different root indices, *u_c_* and *u_d_*, is defined as:

(6) $$\begin{array}{cc} A_{ZC}^{c,d}\left(n,\varepsilon \right) & =\frac{1}{N_{ZC}}\sum_{m=-\infty }^{\infty }x_{ZC}^{c}\left(m\right){\left(x_{ZC}^{d}\left(m-n\right)\right)}^{*}e^{j\frac{2\pi \varepsilon }{N_{ZC}}m}\\ & =\frac{1}{N_{ZC}}\sum_{m=-\infty }^{\infty }x_{u_{c}}\left(m\right)x_{u_{d}}^{*}\left(m-n\right)q\left(m\right)q\left(m-n\right)e^{j\frac{2\pi \varepsilon }{N_{ZC}}m}\\ & =\frac{1}{N_{ZC}}\{\begin{array}{cc} x_{u_{d}}^{*}\left(-n\right)\sum_{m=0}^{N_{ZC}+n-1}x_{u_{cd}}\left(m\right)e^{j\frac{2\pi }{N_{ZC}}\left(nu_{d}+\varepsilon \right)m}, & -N_{ZC}<n\leq 0\\ x_{u_{c}}\left(n\right)e^{j\frac{2\pi }{N_{ZC}}\varepsilon n}\sum_{m=0}^{N_{ZC}-1-n}x_{u_{cd}}\left(m\right)e^{j\frac{2\pi }{N_{ZC}}\left(nu_{c}+\varepsilon \right)m}, & 0<n<N_{ZC} \end{array} \end{array}$$

where *u_cd_* = *u_c_* − *u_d_*. Unlike the AF of RAP 1 in (5), a closed-form expression of (6) cannot be derived for the general values of Doppler shift and *n*. However, when *ε* is an integer value (*ε_I_*), the CAF of RAP 1 can be derived as follows:

(7) $$\begin{array}{cc} A_{ZC}^{c,d}\left(0,\varepsilon_{I}\right) & =\frac{1}{N_{ZC}}\sum_{m=0}^{N_{ZC}-1}x_{u_{cd}}\left(m\right)e^{j\frac{2\pi \varepsilon_{I}}{N_{ZC}}m}\\ & =x_{u_{cd}}^{*}\left(u_{cd}^{-1}\varepsilon_{I}\right)\frac{1}{N_{ZC}}\sum_{m=0}^{N_{ZC}-1}x_{u_{cd}}\left(m+u_{cd}^{-1}\varepsilon_{I}\right)\\ & =x_{u_{cd}}^{*}\left(u_{cd}^{-1}\varepsilon_{I}\right)\frac{1}{N_{ZC}}\sum_{m=0}^{N_{ZC}-1}x_{u_{cd}}\left(m\right)\\ & =x_{u_{cd}}^{*}\left(u_{cd}^{-1}\varepsilon_{I}\right)e^{-j\frac{2\pi u_{cd}\eta \vartheta^{2}}{N_{ZC}}}\{\begin{array}{l} G\left(\eta \left(u_{c}-u_{d}\right),N_{ZC}\right),\;u_{c}>u_{d}\\ G^{*}\left(\eta \left(u_{d}-u_{c}\right),N_{ZC}\right),\;u_{c}<u_{d} \end{array} \end{array}$$

where $u_{cd}^{-1}$ denotes the multiplicative inverse of *u_cd_*. Further, *η* = (*N_ZC_* + 1)/2 and *ϑ* = (*N_ZC_* − 1)/2. The Gauss sum expression, *G*(*a,N*), in (7) is given by:

(8) $$G\left(a,N\right)=\left(\frac{a}{N}\right)\frac{1}{\sqrt{N}}\frac{1-j^{N}}{1-j}$$

where (*a/N*) denotes the Legendre symbol with the value of {−1, 0, 1} and $\left|G\left(a,N\right)\right|=1/\sqrt{N}$.

**Figure 7:**
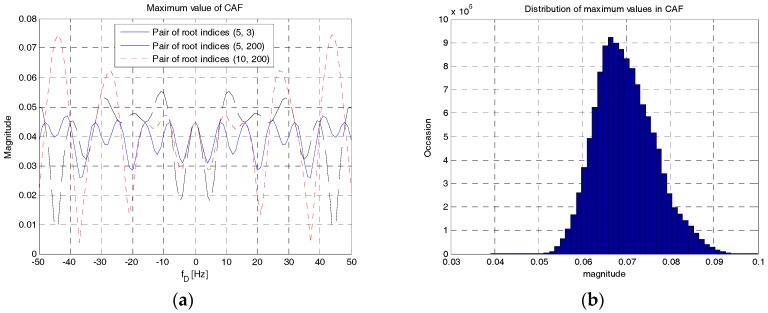
Maximum value of the CAF of RAP 1. (**a**) Doppler domain; (**b**) Distribution.

Figure 7 shows the maximum value of the CAF of RAP 1. In Figure 7a, three pairs of root indices, (5, 3), (5, 200), and (10, 200), are used for the design of RAP 1 and the Doppler shift is changed from −50 Hz to 50 Hz. As shown in this figure, a pair of odd and prime root indices, (3, 5), achieves smaller variation in magnitude. A pair of even root indices, (10, 200), produces higher variation in magnitude. For $\left|f_{D}\right|\leq 50\;\mathrm{Hz}$, the ranges of variation are [0.027, 0.047], [0.01, 0.05], and [0.05, 0.075] when the pairs of root indices, (3, 5), (5, 200), and (10, 200) are used, respectively. However, the ranges of variation are smaller than 0.1 (10%). Figure 7b shows the distribution of the maximum magnitudes evaluated with RAPs by changing the value of the Doppler shift from −50 Hz to 50 Hz with a step size of 1 Hz and the values of root indices of *u_c_* and *u_d_* for all possible combinations. As shown in Figure 7b, the maximum magnitude of CAF is concentrated around 0.07 (7%). Therefore, it is possible to use any root indices in RAP 1 with a cross-correlation level less than 0.1 even in high Doppler environments.

#### 3.2.2. Proposed RAP 2

RAP 2 is generated using a Doppler-insensitive waveform, i.e., the LFM waveform. The information on the ID of a UE is mapped to the parameters of LFM waveform: frequency shift parameter, *f*, and frequency sweeping parameter, *β*. RAP 2 for UE *c* is generated in the continuous-time domain with a symbol duration, *τ*, as follows:

(9) $$\begin{array}{l} x_{LFM}^{c}\left(t\right)=x_{\tau }\left(f_{c},\beta_{c},t\right)p_{\tau }\left(t\right)\\ -B\leq \beta_{c}\leq B,\;\frac{-B+\left|\beta_{c}\right|}{2}\leq f_{c}\leq \frac{B-\left|\beta_{c}\right|}{2} \end{array}$$

where *B* denotes the operational channel bandwidth (4 KHz in UL 0). The LFM waveform, *x_τ_*(*f*,*β*,*t*) and rectangular pulse, *p_τ_*(_t_), are defined as follows:

(10) $$x_{\tau }\left(f,\beta ,t\right)=e^{j\pi \left(2ft+\frac{\beta }{\tau }t^{2}\right)},\;p_{\tau }\left(t\right)=\{\begin{array}{l} \frac{1}{\sqrt{\tau }},\;-\frac{\tau }{2}\leq t\leq \frac{\tau }{2}\\ 0,\;\mathrm{otherwise} \end{array}$$

The AF of RAP 2 with the ID of UE, *c*, is given as follows:

(11) $$\begin{array}{cc} A_{LFM}^{c}\left(t,f_{D}\right) & =\int_{-\infty }^{\infty }x_{LFM}^{c}\left(s\right){\left(x_{LFM}^{c}\left(s-t\right)\right)}^{*}e^{j2\pi f_{D}s}ds\\ & =\{\begin{array}{cc} \varphi_{\tau }^{*}\left(\frac{2f_{c}+f_{D}}{2}\tau t\right)\rho_{LFM}\left(t\right)\mathrm{sinc}\left(\left(f_{D}\tau +\beta t\right)\rho_{LFM}\left(t\right)\right), & -\tau \leq t<\tau \\ \;0, & \mathrm{otherwise} \end{array} \end{array}$$

where $\rho_{LFM}\left(t\right)=1-\left|t\right|/\tau$. The derivation of (11) is given in the Appendix. As evident from (11), the magnitude of $A_{LFM}^{c}\left(t,f_{D}\right)$ varies depending on *τ*, *f_D_* and *β_c_* [16]. However, the parameter *f_c_* does not affect the magnitude of AF. Further, we analyze the effect of AF in (11) on *f_D_* and *β_c_* with the symbol duration being fixed to 125 ms.

**Figure 8:**
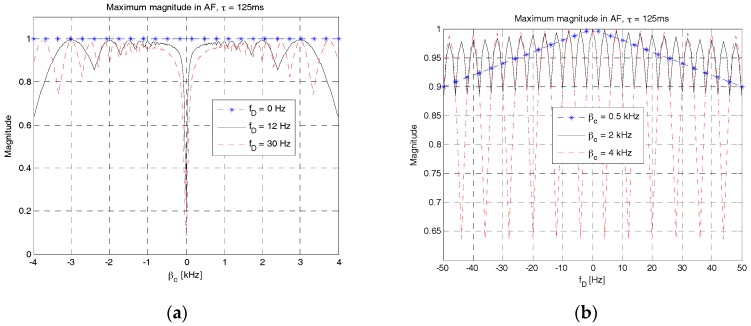

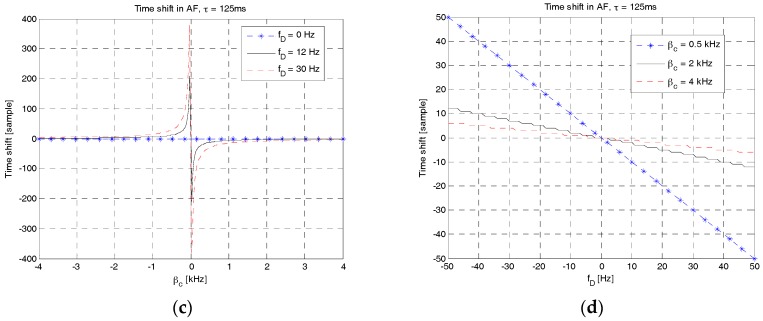
Maximum value of AF of RAP 2. (**a**) Maximum magnitude in *β_c_* domain; (**b**) Maximum magnitude in *f_D_* domain; (**c**) Time shift in *β_c_* domain; (**d**) Time shift in *f_D_* domain.

Figure 8 shows the maximum magnitudes and corresponding time shifts of AF in *β_c_* and *f_D_* domains. Figure 8a,c show the maximum magnitudes and corresponding time shifts of AF in *β_c_* domain for three different values of the Doppler shift, i.e., 0 Hz, 12 Hz and 30 Hz. As shown in these figures, there is no variation in both the magnitude (all 1 s) and time shift (all 0 s) when *f_D_* = 0 Hz. However, magnitude and time shift vary when there is a Doppler shift. The degree of variation is significant especially at a small value of *β_c_*. It can be observed that, when *β_c_* = 0 Hz and *f_D_* = 30 Hz, the maximum value becomes smaller than 0.2 and the absolute value of time shift increases up to 480 samples. With a sampling rate of 4 kHz, the timing error of 480 samples corresponds to 180 m in the sound speed of 1500 m/s. In order to obtain a high detection probability of RAP with a small timing error, the value of *β_c_* should be selected such that high magnitude and small time shift of AF can be achieved. From these figures, the smallest value of *β_c_* (denoted as *β*_0_), i.e., 0.5 kHz, is selected and used in the following simulations. Figure 8b,d show the maximum value and corresponding time shifts of AF in *f_D_* domain for three different values of *β_c_*, i.e., 0.5 kHz, 2 kHz and 4 kHz. As shown in Figure 8b, the maximum value decreases slowly in a triangular manner when *β_c_* = 0.5 kHz, and fluctuates fast when *β_c_* = {2, 4} kHz. However, the time shift varies in a reverse manner as shown in Figure 8d. The time shift in the case of *β_c_* = 0.5 kHz increases faster than that in the cases of 2 kHz and 4 kHz. At the Doppler shift of 50 Hz, the time shift is 50, 12, and 6 samples when the value of *β_c_* is 0.5 kHz, 2 kHz, and 4 kHz, respectively. In summary, RAP 2 is robust to Doppler shifts as long as the frequency sweeping parameter is set to be greater than *β*_0_. The time shift decreases in a Doppler environment as the frequency sweeping parameter increases.

Subsequently, to analyze the cross-correlation properties of the proposed RAP 2 with different IDs of UEs under the effect of time delay and Doppler shift, the CAF of RAP 2 with IDs *c* and *d* is defined as:

(12) $$\begin{array}{cc} A_{LFM}^{c,d}\left(t,f_{D}\right) & =\int_{-\infty }^{\infty }x_{LFM}^{c}\left(s\right){\left(x_{LFM}^{d}\left(s-t\right)\right)}^{*}e^{j2\pi f_{D}s}ds\\ & =\left(\frac{C_{c,d}\left(t,f_{D}\right)}{a_{c,d}\tau }+j\mathrm{sgn}\left(\Delta \beta \right)\frac{S_{c,d}\left(t,f_{D}\right)}{a_{c,d}\tau }\right)x_{\tau }\left(f_{d},-\beta_{d},t\right)e^{-j\pi \Delta \beta \tau {\left(\frac{\Delta f\tau +\beta_{d}t+f_{D}\tau }{\Delta \beta \tau }\right)}^{2}} \end{array}$$

where $-\tau \leq t\leq \tau ,\;\Delta f=f_{c}-f_{d},\;\Delta \beta =\beta_{c}-\beta_{d},\;\Delta \beta \neq 0$, and

(13) $$\begin{array}{l} S_{c,d}\left(t,f_{D}\right)=\{\begin{array}{l} S\left(a_{c,d}s_{t}+b_{c,d}\left(t,f_{D}\right)\right)+S\left(a_{c,d}s_{0}-b_{c,d}\left(t,f_{D}\right)\right),-\tau \leq t<0\\ S\left(a_{c,d}s_{0}+b_{c,d}\left(t,f_{D}\right)\right)+S\left(a_{c,d}s_{t}-b_{c,d}\left(t,f_{D}\right)\right),\;0\leq t<\tau \end{array}\\ C_{c,d}\left(t,f_{D}\right)=\{\begin{array}{l} C\left(a_{c,d}s_{t}+b_{c,d}\left(t,f_{D}\right)\right)+C\left(a_{c,d}s_{0}-b_{c,d}\left(t,f_{D}\right)\right),-\tau \leq t<0\\ C\left(a_{c,d}s_{0}+b_{c,d}\left(t,f_{D}\right)\right)+C\left(a_{c,d}s_{t}-b_{c,d}\left(t,f_{D}\right)\right),\;0\leq t<\tau \end{array}\\ s_{t}=\frac{\tau }{2}-\left|t\right|,a_{c,d}=\sqrt{\frac{\left|\Delta \beta \right|\pi }{\tau }},b_{c,d}\left(t,f_{D}\right)=\left(\frac{\Delta f\tau +\beta_{d}t+f_{D}\tau }{\Delta \beta \tau }\right)\sqrt{\left|\Delta \beta \right|\tau \pi } \end{array}$$

The derivation of (12) is given in the appendix. When *β_c_* = *β_d_*, the CAF of RAP 2 can be derived as:

(14) $$\begin{array}{cc} A_{LFM}^{c,d}\left(t,f_{D}\right) & =\frac{x_{\tau }\left(f_{c},-\beta_{c},t\right)}{\tau }\int_{-\infty }^{\infty }\varphi_{\tau }^{*}\left(\Delta f\tau s+\beta_{c}ts+f_{D}\tau s\right)p_{\tau }\left(s\right)p_{\tau }\left(s-t\right)ds\\ & =\varphi_{\tau }\left(-\frac{f_{c}+\Delta f+f_{D}}{2}\tau t\right)\rho_{\tau }\left(t\right)\mathrm{sinc}\left(\left(\Delta f\tau +f_{D}\tau +\beta_{c}t\right)\rho_{\tau }\left(t\right)\right) \end{array}$$

Since (14) can be viewed as a shifted version of a sinc function, the time shift, $t_{S}^{c,d}$, and the corresponding peak value, ${\tilde{\rho }}_{LFM}^{c,d}$, are expressed as follows:

(15) $$t_{S}^{c,d}=-\frac{\tau }{\beta_{c}}\left(\Delta f+f_{D}\right),{\tilde{\rho }}_{LFM}^{c,d}=\rho_{LFM}\left(t_{S}^{c,d}\right)$$

when *β_c_* is fixed, the time shift increases proportionally with the values of *f_c_*, *f_d_*, and *f_D_*. Moreover, the peak value decreases as the time shift increases. Further, (15) signifies that the time shift should be larger than the symbol duration to avoid time ambiguity, which may lead to false detection, i.e.:

(16) $$\frac{\left|t_{S}^{c,d}\right|}{\tau }\geq 1\Leftrightarrow \left|\Delta f+f_{D}\right|\geq \left|\beta_{c}\right|$$

From (9) and (16), we can obtain the condition of Δ*f* that does not produce time ambiguity, assuming that Δ*f* is much larger than *f_D_*, as follows:

(17) $$\beta_{c}\leq \Delta f\leq \frac{B-\beta_{c}}{2},\;\beta_{c}>0$$

The condition in (17) is satisfied for all the values of *β_c_* less than *B*/3. If *β_c_* is larger than *B*/3, only one RAP can be generated with *β_c_*. Otherwise, time ambiguity will occur. For example, when Δ*f* = *β_c_* = 0.5 kHz, we can generate seven RAPs with $\left|f_{c}\right|=\left\{0,\;0.5,\;1,\;1.5\right\}\;\mathrm{kHz}$ from a single value of the frequency sweeping parameter. In this case, the frequency of RAP after sweeping stays within the channel bandwidth and time ambiguity does not occur.

**Figure 9:**
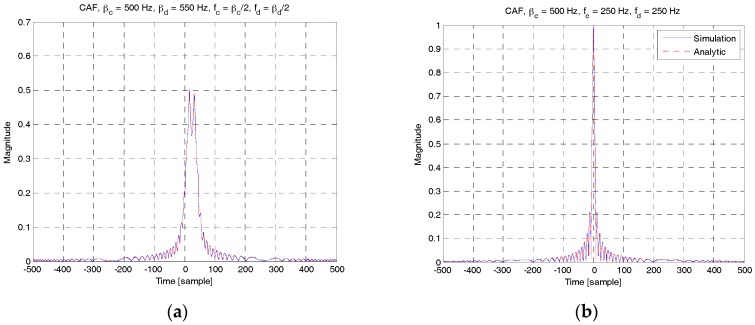
CAF of RAP 2 (analytical vs. simulation). (**a**) $\beta_{c}\neq \beta_{d}$, (12); (**b**) $\beta_{c}=\beta_{d}$, (14).

In Figure 9, the analytical solutions in (12) and (14) are compared with the simulation results when *f_D_* = 0 Hz. The analytical solution in (12) is obtained by numerical evaluation of Fresnel integral whereas the simulation results are obtained through a direct cross-correlation operation between the two LFM waveforms. As shown in this figure, the analytical solutions are consistent with the simulation results (almost indistinguishable). Figure 9a shows the case of Δ*β* ≠ 0. Figure 9b shows a special case when Δ*β* = 0 and Δ*f* = 0. In this case, the highest peak occurs at the correct timing (t=0) because the two RAPs are generated using the same LFM waveform.

**Figure 10:**
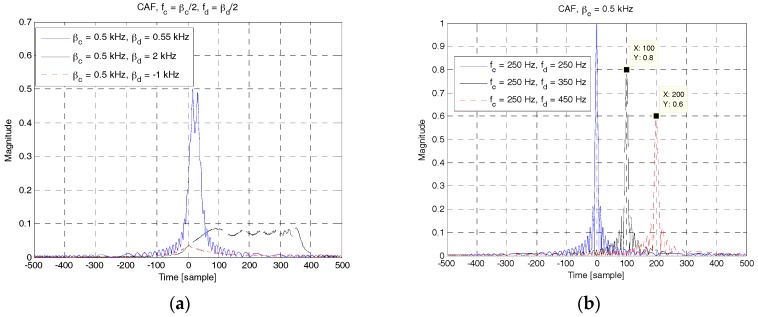
CAF of RAP 2 for different values of β and f. (**a**) $\beta_{c}\neq \beta_{d}$, (12); (**b**) $\beta_{c}=\beta_{d}$, (14).

Figure 10 shows the CAFs of RAP 2 for different values of *β_b_* and *f_b_*. Further, *β_c_* = 0.5 kHz and *f_D_* = 0 Hz. In Figure 10a, the analytical solution in (12) is plotted for three different values of *β_b_*, i.e., 0.55 kHz, 2 kHz, and −1 kHz. As shown in this figure, the cross-correlation level decreases as Δ*β* increases. In Figure 10b, the analytical solution in (14) is plotted for three different values of Δ*f*. As shown in this figure, there are two significant peaks other than the main peak at the origin. The two peaks occur when the two RAPs at *f_b_* = 350 Hz (Δ*f* = 100 Hz) and 450 Hz ((Δ*f* = 200 Hz) are received and correlated with an RAP at 250 Hz. This may produce ambiguity in detecting the ID of the UE and time delay. The peak value and the corresponding time delay can be predicted using (15).

The value of Δ*β* should be carefully chosen to have small correlation among RAPs while supporting a large number of RAPs. In order to determine the number of possible RAPs generated by the LFM waveform, we will derive the upper bound of CAF of RAP 2. Here, we define *α_C_* and *α_S_* as the maximum values of cosine and sine Fresnel integrals, respectively. Subsequently, we can obtain the following relationships:

(18) $$\begin{array}{ll} i) & -\alpha_{C}\leq C\left(t\right)\leq \alpha_{C},-\alpha_{S}\leq S\left(t\right)\leq \alpha_{S}\\ \mathrm{ii}) & -2\alpha_{C}\leq C\left(t_{1}\right)+C\left(t_{2}\right)\leq 2\alpha_{C},\;-2\alpha_{S}\leq S\left(t_{1}\right)+S\left(t_{2}\right)\leq 2\alpha_{S}\\ \mathrm{iii}) & 0\leq C_{c,d}^{2}\left(t,f_{D}\right)\leq 4\alpha_{C}^{2},0\leq S_{c,d}^{2}\left(t,f_{D}\right)\leq 4\alpha_{S}^{2}\\ \mathrm{iv}) & 0\leq C_{c,d}^{2}\left(t,f_{D}\right)+S_{c,d}^{2}\left(t,f_{D}\right)\leq 4\left(\alpha_{C}^{2}+\alpha_{S}^{2}\right)\\ v) & 0\leq \frac{C_{c,d}^{2}\left(t,f_{D}\right)+S_{c,d}^{2}\left(t,f_{D}\right)}{a_{c,d}^{2}\tau^{2}}\leq \frac{4\left(\alpha_{C}^{2}+\alpha_{S}^{2}\right)}{a_{c,d}^{2}\tau^{2}}\\ \mathrm{vi}) & 0\;\leq \left|A_{LFM}^{c,d}\left(t,f_{D}\right)\right|\leq \frac{2}{a_{c,d}^{2}\tau }\sqrt{\alpha_{C}^{2}+\alpha_{S}^{2}} \end{array}$$

From (18), we can obtain the upper bound of the CAF of RAP 2 as follows:

(19) $$\Gamma \left(\tau ,\Delta \beta \right)=2\sqrt{\frac{\alpha_{C}^{2}+\alpha_{S}^{2}}{\Delta \beta \tau \pi }},\;\Delta \beta >0$$

From (14), we can also obtain *α_S_* = 0.8948 and *α_C_* = 0.9775 by numerical evaluation of the Fresnel integral with a sampling rate of 4 kHz. As evident from (19), the upper bound value depends on neither the frequency shift *f_c_* nor the Doppler shift *f_D_*, indicating that the values of *f_c_* and *f_D_* do not have a significant impact on the magnitude of CAF. The symbol duration (*τ*) and separation value (Δ*β*) between *β_c_* and *β_d_* are crucial. The larger the value of Δ*βτ*, the smaller the value of the upper bound. From (11), we can observe that the range of frequency sweeping parameter is the union of the negative set, S*_N_*, and positive set, S*_P_*, i.e., $\beta \in \left\{S_{N}\cup^{\text{​}}S_{P}\right\}$ where S*_N_* = [−*B*, −*β*_0_] and S*_P_* = [*β*_0_, *B*]. As discussed in the AF of RAP 2, *β*_0_ denotes the smallest value of *β* that provides a good AF in term of time shift and maximum magnitude value.

**Figure 11:**
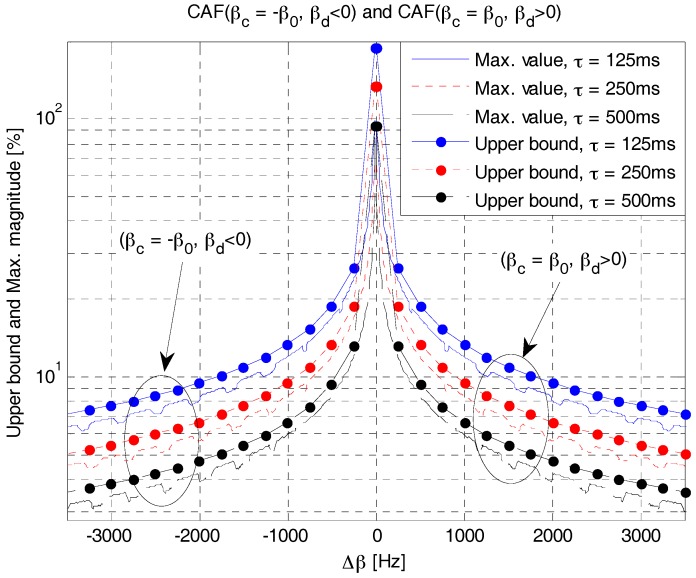
Upper bound and maximum value of CAF of RAP 2.

Figure 11 shows the upper bound given in (19) and the maximum value of CAF of RAP 2 obtained through simulation. Here, *β*_0_ is set to 0.5 kHz and *β_d_*(*β_c_* + Δ*β*) is a variable obtained by changing the value of Δ*β*. As shown in the figure, the upper bound value decreases with the increase of *τ* or Δ*β*. The maximum value of CAF is slightly smaller than the upper bound and decreases with the increase of *τ* or Δ*β*. The upper bound value and maximum magnitude are symmetric about the origin because they both depend only on the parameters (Δ*β*, *τ*). On the positive side of Δ*β*, *β_c_* is set to *β*_0_ and *β_d_* is a variable selected from the set S*_P_*, whereas *β_c_* = −*β*_0_ and *β_d_* ∈ S*_N_* on the negative side of Δ*β*.

**Figure 12:**
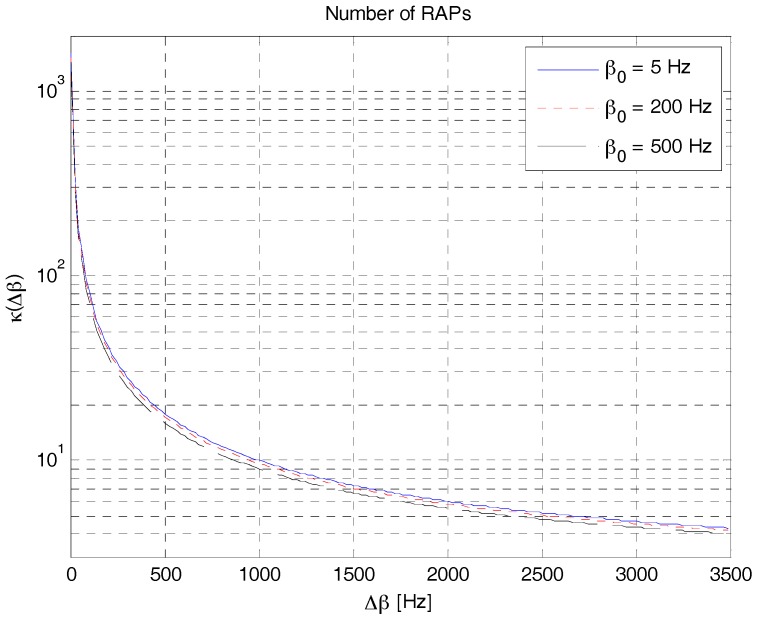
Number of possible RAPs in RAP 2.

Figure 12 shows the number of possible RAPs in RAP 2, obtained using (20), when the value of Δ*β* varies for three different values of *β*_0_. As shown in this figure, the number of possible RAPs decreases with the increase of Δ*β*. For example, the number of possible RAPs is approximately 16 when Δ*β* is 0.5 kHz. In this case (Δ*β* = 0.5 kHz), the maximum value of CAF can be obtained from Figure 9 as 16%, 11%, and 7.5% for symbol durations of 125, 25 and 50 ms, respectively. Notably, the number of possible RAPs in (20) or Figure 10 corresponds to the case where only Δ*β* is considered. The number of RAPs increases when the frequency shift parameter is also considered. If we re-calculate the number of RAPs by considering Δ*f* = *β_c_* ≤ *B*/3 in (21) with the same value of *β*_0_ (0.5 kHz), we can obtain eight additional RAPs. The first six RAPs are generated with *β_c_* = 0.5 kHz and $\left|f_{c}\right|\in \left\{0.5,\;1,\;1.5\right\}\;\mathrm{kHz}$, and the remaining two RAPs are generated with *β_c_* = 1 kHz and $\left|f_{c}\right|=1\;\mathrm{kHz}$. In total, we can obtain 24 different RAPs when Δ*β* = 0.5 kHz.

The number of RAPs that can be generated from an LFM waveform is given by:

(20) $$\kappa \left(\Delta \beta \right)=2\left(\frac{B-\beta_{0}}{\Delta \beta }+1\right)$$

where Δ*β* can be determined from either Figure 9 or the upper bound in (19) as follows:

(21) $$\Delta \beta =4\frac{\alpha_{C}^{2}+\alpha_{S}^{2}}{\tau \pi \Gamma^{2}\left(\tau ,\Delta \beta \right)},\Gamma^{2}\left(\tau ,\Delta \beta \right)>0$$

In summary, the cross-correlation level in the CAF of RAP 2 varies depending on the selection of Δ*β* and *τ*. By increasing the values of Δ*β* and *τ*, we can reduce the cross-correlation level between the RAPs. However, the number of possible RAPs decreases with the increase of Δ*β*. Therefore, when RAP 2 is designed, there is a trade-off between the cross-correlation level and number of RAPs in selecting the value of Δ*β*.

## 4. RAP Detection

In this section, we analyze the detection probability and false alarm probability of the proposed RAPs in a Doppler environment. Here, we assume that the RAP detector is composed of a bank of correlators that correlate/match a received signal with a bank of local RAPs in parallel. The received signal at the input of each correlator, *y*(*t*), is modeled as:

(22) $$y\left(t\right)=\{\begin{array}{ll} {\tilde{z}}^{c}\left(t\right), & H_{0}\\ {\tilde{x}}^{c}\left(t\right)+{\tilde{z}}^{c}\left(t\right), & H_{1} \end{array}$$

where ${\tilde{x}}^{c}\left(t\right)$ denotes the received signal from a desired UE *c*. The interference signals from other UEs and additive noise are given by:

(23) $${\tilde{z}}^{c}\left(t\right)=\sum_{i=0,i\neq c}^{C-1}{\tilde{x}}^{i}\left(t\right)+z\left(t\right)$$

The hypothesis H_1_ refers to the case where the received signal includes the RAP from the desired UE *c*. In the hypothesis H_0_ (null hypothesis), the received signal is composed of all the RAPs received from *C* − 1 UEs except UE *c* and additive noise, *z*(*t*)*.* Here, *C* denotes the number of UEs transmitting RAPs simultaneously. The output of the correlator for UE *c* can be expressed as follows:

(24) $$r^{c}\left(t\right)=\int_{-\tau /2}^{\tau /2}y\left(s+t+\tau /2\right){\left(x^{c}\left(s\right)\right)}^{*}ds$$

where *x^c^*(*t*) denotes the reference RAP transmitted from UE *c*, which can be either RAP 1 or RAP 2. The desired RAP is detected when the correlation output exceeds a threshold value as follows:

(25) $${\hat{t}}^{c}=\underset{t\in \left[0,T_{W}\right]}{\arg\;\max}{\left|r^{c}\left(t\right)\right|}^{2}>\gamma$$

where ${\hat{t}}^{c}$ denotes the estimated time delay of the *c*th UE. Here, *T_W_* denotes the length of the correlator window.

The squared magnitude of each correlator output is given by a random variable with a 2-degree-of-freedom non-chi square density function as follows:

(26) $$f_{R\left[t;H\right]}\left(r\right)=\frac{1}{\sigma^{2}\left[t;H\right]}e^{-\frac{r+\lambda \left[t;H\right]}{\sigma^{2}\left[t;H\right]}}I_{0}\left(\frac{2\sqrt{\lambda \left[t;H\right]r}}{\sigma^{2}\left[t;H\right]}\right)$$

where:

(27) R[t;H]=XRe2[t;H]+XIm2[t;H]σ2[t;H]=2V{XRe[t;H]}=2V{XIm[t;H]}λ[t;H]=E2{XRe[t;H]}+E2{XIm[t;H]}

where *X*_Re_[*t*;H] and *X*_Im_[*t*;H] denote random variables of the real and imaginary parts of the correlator output, given in (24), at time instant *t* with hypothesis H, respectively. Further, *I*_0_(*r*) denotes the first-order Bessel function of the first kind with an argument of *r*. Furthermore, E{·} and V{·} denote the expectation and variance operator, respectively. The detection probability, *P_D_*, and false alarm probability, *P_F_*, are defined as:

(28) $$\begin{array}{l} P_{D}=\mathrm{Pr}\{\mathrm{one}\;\mathrm{correlator}\;\mathrm{output}\;\mathrm{under}\;H_{1}\;\mathrm{is}\;\mathrm{greater}\;\mathrm{than}\;\gamma \}\\ P_{F}=\mathrm{Pr}\{\mathrm{one}\;\mathrm{correlator}\;\mathrm{output}\;\mathrm{under}\;H_{0}\;\mathrm{is}\;\mathrm{greater}\;\mathrm{than}\;\gamma \} \end{array}$$

Although it is not shown in (28) owing to notational difficulty, the time delay can be estimated when the squared magnitude of correlator output exceeds the threshold value. A high peak will appear at the time index where the received signal and reference waveform are fully matched, producing the estimate of time delay. Using the definition in (28), we can obtain the detection probability and false alarm probability as follows [17]:

(29) $$\begin{array}{l} P_{D}=1-\prod_{t=0}^{T_{W}-t_{s}}\mathrm{Pr}\left\{R\left[t;H_{1}\right]\leq \gamma \right\}=1-\prod_{t=0}^{T_{W}-t_{s}}F_{R\left[t;H_{1}\right]}\left(\gamma \right)\\ P_{F}=1-\prod_{t=0}^{T_{W}-t_{s}}\mathrm{Pr}\left\{R\left[t;H_{0}\right]\leq \gamma \right\}=1-\prod_{t=0}^{T_{W}-t_{s}}F_{R\left[t;H_{0}\right]}\left(\gamma \right) \end{array}$$

where:

(30) $$\begin{array}{l} F_{R\left[t;H_{1}\right]}\left(\gamma \right)=\int_{0}^{\gamma }f_{R\left[t;H_{1}\right]}\left(r\right)dr=1-Q_{1}\left(\frac{\sqrt{2\lambda \left[t;H_{1}\right]}}{\sigma \left[t;H_{1}\right]},\frac{\sqrt{2\gamma }}{\sigma \left[t;H_{1}\right]}\right)\\ F_{R\left[t;H_{0}\right]}\left(\gamma \right)=\int_{0}^{\gamma }f_{R\left[t;H_{0}\right]}\left(n\right)dr=1-Q_{1}\left(\frac{\sqrt{2\lambda \left[t;H_{0}\right]}}{\sigma \left[t;H_{0}\right]},\frac{\sqrt{2\gamma }}{\sigma \left[t;H_{0}\right]}\right) \end{array}$$

where *Q*_1_(*a,b*) denotes the first-order Marcum function with the arguments of *a* and *b*. Since the symbol duration of RAP is usually smaller than the correlation window, the output signal in the early part of RAP detector follows the central chi square distribution. In this case, the Marcum function becomes an exponential function as follows [18]:

(31) $$F_{R\left[t;H\right]}\left(\gamma \right)=1-\{\begin{array}{ll} e^{-\frac{\gamma }{\sigma^{2}\left[t;H\right]}}, & \lambda \left[t;H\right]=0\\ Q_{1}\left(\frac{\sqrt{2\lambda \left[t;H\right]}}{\sigma \left[t;H\right]},\frac{\sqrt{2\gamma }}{\sigma \left[t;H\right]}\right), & \lambda \left[t;H\right]\neq 0 \end{array}$$

From (22) and (27), we can obtain the non-centrality parameter as follows:

(32) λc[t;H0]=(∑i=0,i≠cC−1E{Re{Rxi,c(t)}})2+(∑i=0,i≠cC−1E{Im{Rxi,c(t)}})2λc[t;H1]=(∑i=0C−1E{Re{Rxi,c(t)}})2+(∑i=0C−1E{Im{Rxi,c(t)}})2

where $R_{x}^{1,c}\left(t\right)$ denotes a random variable after the cross-correlation between the RAP received from the *i-*th UE and the reference RAP transmitted from UE *c* at the instant *t*. Here, Re{·} and Im{·} denote the real and imaginary parts, respectively.

Subsequently, the detection probability and false alarm probability of the proposed RAPs (RAP 1 and RAP 2) are compared using the analytical and simulation results in a Doppler environment. Here, the window size, *T_W_*, is set to a value twice the symbol duration. The analytical result is obtained using (29) with parameters determined under the assumption of an additive white Gaussian noise (AWGN) channel. The signal-to-noise ratio (SNR) and duration of the RAP are set to −10 dB and 125 ms, respectively. The Marcum function is evaluated numerically using MATLAB building functions. The sampling rate is set to the same value (4 kHz) as the bandwidth of UL 0.

**Figure 13:**
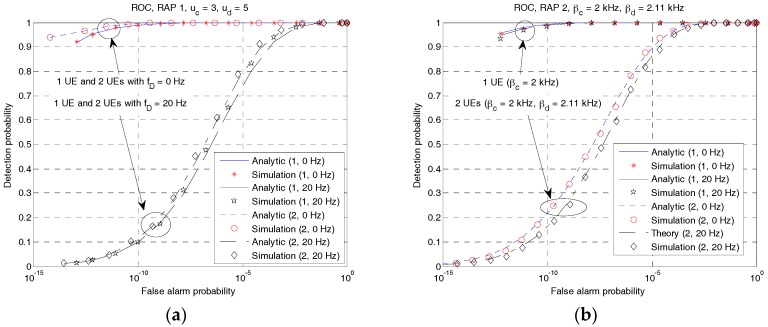
ROC curves (analytical vs. simulation). (**a**) RAP 1; (**b**) RAP 2.

Figure 13 compares the receiver operating characteristic (ROC) curves of the proposed RAPs. For RAP 1, the first and second RAPs are generated using ZC sequences with root indices of 3 and 5 (i.e., *u_c_* = 3, *u_d_* = 5). For RAP 2, the first and second RAP are generated using LFM waveforms with the frequency sweeping parameters of 2 kHz and 2.11 kHz (Δ*β* = *β_c_* − *β_d_* = 110 Hz), respectively. In RAP 2, the frequency shifting parameter is set to half of *β_c_*, i.e., *f_c_* = *β_c_*/2 and *f_d_* = *β_d_*/2. Figure 13a,b show the ROC curves (detection probability versus false alarm probability) of RAP 1 and RAP 2, respectively. As shown in these figures, the simulation results are consistent with the analytical results in all the cases. Here, the first argument in the legend represents the number of UEs. The value ‘1’ represents the case where there is no interfering UE. There is an interfering UE in the case of ‘2’. As shown in Figure 13a, performance degradation caused by the interfering UE is small, indicating that RAP 1 is robust against the interference. Notably, the maximum magnitude of CAF for a pair of root indices of 3 and 5 is small (5.5%), as shown in Figure 7a. However, when there is a Doppler frequency, the performance of RAP 1 decreases significantly owing to its Doppler sensitivity. As shown in Figure 13a, the detection probability is 1 at the false alarm probability of 10^−9^ when *f_D_* = 0 Hz, and 0.2 when *f_D_* = 20 Hz. However, RAP 2 is robust against Doppler shift, as shown in Figure 13b. For the case of a single UE, the ROC curve at *f_D_* = 20 Hz is almost the same as that at *f_D_* = 0. However, the performance of RAP 2 decreases significantly when an interfering UE exists. As shown in Figure 13b, the detection probability is close to 1 at the false alarm probability of 10^−9^ for the case of a single UE and 0.3 for the case of two UEs.

**Figure 14:**
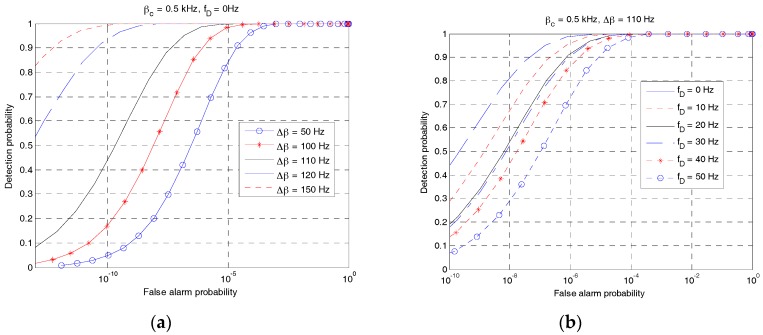
ROC curves of RAP 2 when two UEs exist. (**a**) Different Δ*β*; (**b**) Different *f_D_*.

Figure 14 shows the ROC curves of RAP 2 for different values of Δ*β* and *f_D_* when two UEs exist. Here, the value of Δ*β* is changed from 50 Hz to 150 Hz, when *f_D_* = 0 Hz, *β_c_* = 0.5 kHz, and *β_d_* = *β_c_* + Δ*β*. As shown in Figure 14a, the detection probability increases with the increase of Δ*β*. Notably, the maximum value of CAF of the LFM waveform decreases with the increase of Δ*β*, as shown in Figure 9. Figure 14b shows the ROC curves of RAP 2 for different values of Doppler shift when Δ*β* = 110 Hz. As expected, the detection probability decreases with the increase of Doppler shift.

The distance can be estimated from the estimated value of time delay as follows:

(33) $$\hat{d}=v_{c}\hat{t}$$

**Figure 15:**
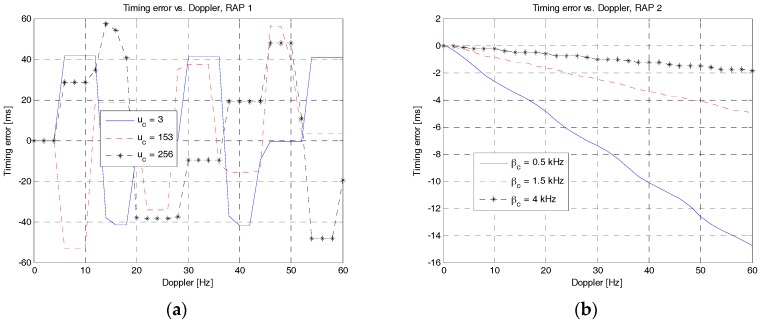
Estimation error of time delay in a Doppler environment. (**a**) RAP 1; (**b**) RAP 2.

The time delay, $\hat{t}$, can be estimated using (25). Here, it is assumed that the speed of sound is constant although the speed is known to depend on the water properties of temperature, salinity, and pressure. Figure 15 compares the performances of time delay estimation in a Doppler environment when RAP 1 and RAP 2 are used. As shown in Figure 15a, the timing error of RAP 1 with three different values of root indices is up to 60 ms when there is a Doppler shift. On the other hand, the timing error of RAP 2 with three different values of *β_c_* is up to 15 ms, which is much smaller than that of RAP 1, as shown in Figure 15b. In terms of distance errors, RAP 1 and RAP 2 can result in errors up to 90 m (60 × 1.5) and 22.5 m (15 × 1.5), respectively.

Based on the analytical and simulation results in this section, RAP 2 is selected for our experiment, because RAP 2 is robust to Doppler shift. Although the detection probability of RAP 2 decreases in a multi-UE environment, it can be increased to a certain extent by selecting an appropriate value of Δ*β*. The timing error can also be reduced to a certain extent with an appropriate value of Δ*β*.

## 5. Experimental Results

In 2015, a new research project on underwater acoustic communication systems was launched in Korea. Its ultimate goal was to deploy an underwater acoustic cellular system connected to a terrestrial cellular network (LTE), as shown in Figure 1. The project consisted of three phases: the development of Link 1 and Link 2, and network interworking (terrestrial cellular network and underwater acoustic cellular network). A field experiment was performed on 8 December 2016. The experimental site is located near Geoje Island in the south sea of the Korean peninsula. Based on the analytical and simulation results in the previous sections, RAP 2 was selected for random access (UL 0) in the initialization stage. In the experiment, the RAP was generated by a signal generator (National Instrument, Austin, TX, USA). Amplifier, transducer, analog filter, digital filter, hydrophone, ADC/DAC, and Zynq-7100 Chipset board (Xilinx, San Jose, CA, USA) were designed for the experiment.

Although various transmission/multiplexing schemes (single carrier, OFDM, CDMA, and SC-FDMA) were investigated in this project for performance comparison, an OFDM-based system was considered in this paper. The OFDM parameters of carrier frequency, bandwidth, subcarrier spacing, and FFT size were set to 32 kHz, 4 kHz, 9.765 Hz, and 512, respectively. The number of used subcarriers and effective bandwidth were 498 and 4.863 kHz, respectively. The OFDM symbol length, CP length, and sampling rate were 125 ms, 22.6 ms, and 5 kHz, respectively. Using these parameters, the data transmission and downlink synchronization parts were successfully completed using a transducer, hydrophone, and underwater modem, which were designed for this project.

In this paper, we focus on the performance verification of RAP 2 in terms of AF and CAF. Here, four RAPs with frequency sweeping parameters of 0.5, 1.5, 2.5 and 3.7 kHz were transmitted from a UE and received at a UBS with distances of 1, 2, 3 and 4 km, respectively. The transmitted power was 23 W and the symbol duration of each RAP was 750 ms. The frequency shift parameter was set to half of the frequency sweeping parameter, except in the case of RAP 2 generated at 3.7 kHz to avoid shifting out of the operational bandwidth.

**Figure 16:**
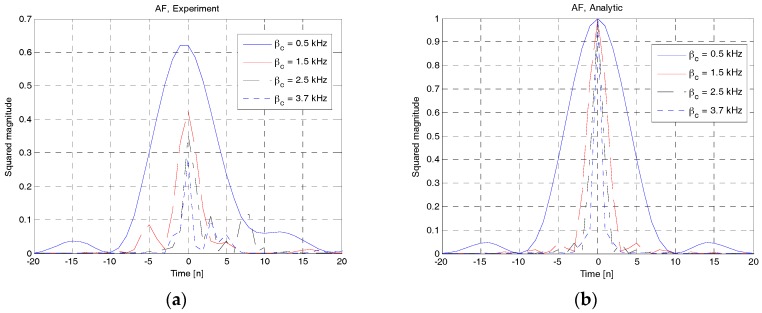
AF comparison (experiment vs. analytical). (**a**) Experiment; (**b**) Analytical.

Figure 16a,b show the squared magnitudes of the AF obtained from the experimental and analytical results, respectively. Notably, the analytical solution in (11) was obtained in an ideal condition (channels and noise were ignored) whereas the experimental results were obtained in underwater channels. For experimental results, the timing index corresponding to the maximum value of LFM correlator for RAP detection was normalized to 0. Notably, the LFM peak usually occurred at the strongest multipath component. As shown in Figure 14a, the four curves obtained from experimental data have different squared magnitudes because the distances between UE and UBS vary depending on the value of the frequency sweeping parameter (*β_c_*). However, the curves obtained using the analytical solution have the same squared magnitude because pathloss has not been considered. As evident from the analytical results in Figure 16b, the highest peak occurs at zero timing index and the autocorrelation function decreases in a symmetric fashion. The autocorrelation function becomes sharper as *β_c_* increases. By comparing the experimental and analytical results, we can observe that the AFs of RAPs in Figure 16a are not symmetric but distorted by the effects of channels and noise. However, both the results are of the same shape except for a small difference owing to channels and noise.

**Figure 17:**
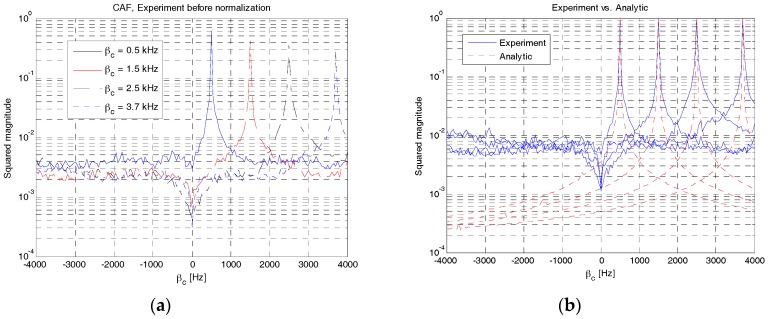
CAF comparison (experiment vs. analytical). (**a**) Experiment before normalization; (**b**) Experiment vs. Analytical.

Figure 17 shows the maximum squared magnitude values of CAF obtained from experimental and analytical results. Here, the received signal is correlated with all possible RAPs generated locally with Δ*β* of 50 Hz. As shown in Figure 17a, four distinct peaks occur at the value of *β_c_* (0.5, 1.5, 2.5 and 3.7 kHz) where the received waveforms are matched with the local counterparts. The four peak values decrease owing to pathloss. The four peaks are well-separated, thus facilitating a successful detection of each RAP. In Figure 17b, the normalized versions of CAF in Figure 17a are compared with the analytical results. As shown in Figure 17b, the analytical result also produces four distinct peaks at the same position. However, the analytical results are sharper and have lower cross-correlation levels than the experimental results because the channels and noise are ignored in analytical results. In summary, the AF and CAF analytically obtained in Section 3 are shown to be similar to those obtained from experimental data, except for a small difference in magnitude owing to the effect of channels and noise. Therefore, the upper bound of CAF and the number of RAPs derived in this paper can be effectively used to select the parameters of RAP 2. In this experiment, the following parameters are selected: *β_c_* = {0.5, 1.5, 2.5} kHz, *β_d_* = {1.5, 2.5, 3.7} kHz, and Δ*β* = {−1, −1, −1.2} kHz. In this case, the upper bound of CAF, given in (19), is {5.46, 5.46, 4.98}%. The numbers of RAPs, given in (20), are 9 and 7 when the upper bound values are 5.46% and 4.98%, respectively. Six additional RAPs are generated using different frequency shifting parameters ($\left|f_{c}\right|\in \left\{0.5,\;1,\;1.5\right\}\;\mathrm{kHz}$).

## 6. Conclusions

In this paper, two different types of RAPs (RAP 1 and RAP 2) were proposed to detect the identity of UE and estimate the time delay between a UBS and a UE at the physical layer, after showing that the conventional RAP used in LTE systems is not appropriate for underwater acoustic cellular systems. The properties of RAP 1 and RAP 2 are evaluated using analytical solution and computer simulation, and can be summarized as follows. The AF and CAF of RAP 1 have desirable properties when there is no Doppler shift. RAP 1 provides a sharp peak around the origin in the AF and a small cross-correlation level in the CAF when there is no Doppler shift. The performance degradation (detection probability) caused by an interfering UE is minimal. However, undesired properties develop in AF when there is a Doppler shift. The magnitude of AF at the correct timing is reduced and the side peaks increase when there is a Doppler shift. The detection probability of RAP 1 also decreases significantly owing to its Doppler sensitivity. However, RAP 2 is robust to Doppler shift because it is generated using a Doppler-insensitive waveform. The detection probability of RAP 2 is degraded slightly when there is a Doppler shift. However, the magnitude of AF in RAP 2 varies depending on the selection of *β_c_*. The number of possible RAPs and the cross-correlation level of CAF vary depending on the selection of Δ*β*. In this paper, analytical solutions for the upper bound of the cross-correlation level and the number of possible RAPs are provided such that they can be used in selecting the appropriate values of *β_c_* and Δ*β*, required for the design of RAP 2. Finally, the AF and CAF analytically obtained in this paper are shown to be similar to those obtained using experimental data, except for a small difference caused by channels and noise. 

## Appendix

Some useful properties, which will be used to simplify the algebraic equations related to the LFM waveform, are given as follows:

(A1) $$\begin{array}{l} x_{\tau }\left(f,\beta ,t\right)=e^{-j\pi \beta \tau {\left(f/\beta \right)}^{2}}e^{j\mathrm{sgn}\left(\beta \right){\left(at+b\right)}^{2}},\;\beta \neq 0\\ \;x_{\tau }^{*}\left(f,\beta ,t\right)=x_{\tau }\left(-f,-\beta ,t\right)\\ \;x_{\tau }\left(f,\beta ,s-t\right)=x_{\tau }\left(-f,\beta ,t\right)x_{\tau }\left(f,\beta ,s\right)\varphi_{\tau }\left(\beta st\right)\\ \;x_{\tau }\left(f_{1},\beta_{1},t\right)x_{\tau }\left(f_{2},\beta_{2},t\right)=x_{\tau }\left(f_{1}+f_{2},\beta_{1}+\beta_{2},t\right) \end{array}$$

where sgn(*β*) denotes the signum function with the values of {−1, 0, 1} for negative, zero, and positive values of *β*, respectively. Furthermore:

(A2) $$\varphi_{\tau }\left(t\right)=e^{-j\frac{2\pi }{\tau }t},\;a=\sqrt{\frac{\left|\beta \right|\pi }{\tau }},\;b=\sqrt{\left|\beta \right|\tau \pi }\frac{f}{\beta }$$

Additional integral functions, which will be used to simplify the algebraic equations in this sub-section, are given as follows:

(A3) $$\begin{array}{l} \int_{t_{1}}^{t_{2}}\sin{\left(as+b\right)}^{2}ds=\frac{S\left(at_{2}+b\right)-S\left(at_{1}+b\right)}{a}\\ \int_{t_{1}}^{t_{2}}\cos{\left(as+b\right)}^{2}ds=\frac{C\left(at_{2}+b\right)-C\left(at_{1}+b\right)}{a} \end{array}$$

where $S\left(t\right)=\sqrt{\pi /2}S_{n}(\sqrt{2/\pi }t$) and $C\left(t\right)=\;\sqrt{\pi /2}C_{n}(\sqrt{2/\pi }t$) denote the sine and cosine Fresnel integrals, respectively. The normalized forms of Fresnel integrals are given by:

(A4) $$S_{n}\left(t\right)=\int_{0}^{t}\sin\left(\frac{\pi s^{2}}{2}\right)ds,\;C_{n}\left(t\right)=\int_{0}^{t}\cos\left(\frac{\pi s^{2}}{2}\right)ds$$

The AF and CAF of RAP 2 are derived using (A1) and (A3) as follows:

(A5) $$\begin{array}{ll} A_{LFM}^{c}\left(t,f_{D}\right) & =\int_{-\infty }^{\infty }x_{\tau }\left(f_{c},\beta_{c},s\right)x_{\tau }^{*}\left(f_{c},\beta_{c},s-t\right)e^{j2\pi f_{D}s}p_{\tau }\left(s\right)p_{\tau }\left(s-t\right)ds\\ & =\frac{x_{\tau }\left(f_{c},-\beta_{c},t\right)}{\tau }\{\begin{array}{l} \int_{-\tau /2}^{\tau /2+t}e^{j\frac{2\pi \left(\beta_{c}t+f_{D}\tau \right)}{\tau }s}ds,\;-\tau \leq t<0\\ \int_{-\tau /2+t}^{\tau /2}e^{j\frac{2\pi \left(\beta_{c}t+f_{D}\tau \right)}{\tau }s}ds,\;0\leq t<\tau \end{array} \end{array}$$

(A6) $$\begin{array}{ll} A_{LFM}^{c,d}\left(t,f_{D}\right) & =\int_{-\infty }^{\infty }x_{\tau }\left(f_{c},\beta_{c},s\right)x_{\tau }^{*}\left(f_{d},\beta_{d},s-t\right)e^{j2\pi f_{D}s}p_{\tau }\left(s\right)p_{\tau }\left(s-t\right)ds\\ & =\frac{x_{\tau }\left(f_{d},-\beta_{d},t\right)}{\tau }\int_{-\infty }^{\infty }x_{\tau }\left(\Delta f+\frac{\beta_{d}t}{\tau }+f_{D},\Delta \beta ,s\right)p_{\tau }\left(s\right)p_{\tau }\left(s-t\right)ds \end{array}$$

Equation (A6) yields (12) when Δ*β* ≠ 0 and (14) when Δ*β* = 0.
